# Accurate MP2-based force fields predict hydration free energies for simple alkanes and alcohols in good agreement with experiments

**DOI:** 10.1063/5.0035032

**Published:** 2020-12-28

**Authors:** T. Ryan Rogers, Feng Wang

**Affiliations:** Department of Chemistry and Biochemistry, University of Arkansas, Fayetteville, Arkansas 72701, USA

## Abstract

Force fields for four small molecules, methane, ethane, methanol, and ethanol, were created by force matching MP2 gradients computed with triple-zeta-quality basis sets using the Adaptive Force Matching method. Without fitting to any experimental properties, the force fields created were able to predict hydration free energies, enthalpies of hydration, and diffusion constants in excellent agreements with experiments. The root mean square error for the predicted hydration free energies is within 1 kJ/mol of experimental measurements of Ben-Naim *et al.* [J. Chem. Phys. **81**(4), 2016–2027 (1984)]. The good prediction of hydration free energies is particularly noteworthy, as it is an important fundamental property. Similar hydration free energies of ethane relative to methane and of ethanol relative to methanol are attributed to a near cancellation of cavitation penalty and favorable contributions from dispersion and Coulombic interactions as a result of the additional methyl group.

## I. INTRODUCTION

Computer simulations of molecular systems are now routinely utilized in many scientific disciplines. Since quantum mechanical (QM) simulations are computationally intensive for systems with thousands of atoms or more, molecular mechanics (MM) force fields (FFs) are an indispensable tool in molecular simulations. Force field development is, therefore, of essential importance, especially for studying complex systems over extended time scales.^1–4^

The development of some of the most popular FFs has relied on a mix of experimental and *ab initio* data. For example, the Assisted Model Building with Energy Refinement (AMBER)^5^ and Optimized Potentials for Liquid Simulations, All-Atom (OPLS-AA)^6^ rely on experimental data for bonds and angles and use mostly QM data for dihedral parameters.^7^ While AMBER and Chemistry at Harvard Molecular Mechanics (CHARMM) obtain partial charges based on QM, OPLS-AA and Groningen Molecular Simulation (GROMOS) model^8^ determine charges mostly by fitting to liquid properties of pure solvents.^7^ In each of these FFs, Lennard-Jones (LJ) functions are used to model the nonbonded short-range interactions, and such parameters are typically fit to reproduce experimental densities and vaporization enthalpies of pure liquids.^7^ When intermolecular parameters are obtained from pure components, solute–solvent interactions are usually obtained from combination rules; thus, the accuracy of solution phase properties relies heavily on the quality of the combination rules employed.

For a solution, the free energy of solvation is one of the most important properties of the solute. The solvation free energy (SFE) is directly related to many fundamental properties, such as partition coefficients, solubilities, and vapor pressures.^9–12^ Given the importance of SFE, this quantity is often a focus when developing FFs.^13^ Some FFs have been directly fitted to recover SFEs.^8,14–16^ When SFEs are not fitted, the ability to recover SFEs is frequently used as a benchmark to assess the quality of the FF.^6^

Not surprisingly, emphasis on solution phase properties during the FF development has led to advancements in the quality of predicted SFEs. The new OPLS FF has shown improvements by including the so-called bond charge correction terms,^17,18^ parameterized, in part, to reproduce SFEs.^19^ Inclusion of polarization effects in the charge models during free energy perturbation (FEP) computation has been shown to improve agreement with experiments.^20,21^ Recently, RESP2 has been developed to incorporate condensed phase effects on charges,^22^ and Fennell *et al.* have shown improved SFE predictions by parameterizing charges and LJ parameters to better model the solution phase dielectric response.^23^ However, in order for FFs to reproduce experimental SFEs within 1 kcal/mol, it has been suggested that proper modeling of short-range nonbonded interactions must be a focus during FF development.^24^ Indeed, improved accuracy has been observed by tailoring the solute–solvent interactions instead of relying on combination rules.^25–27^

In this work, we use the Adaptive Force Matching (AFM) methods^28,29^ to develop FFs for predicting SFEs in an aqueous solution, also known as hydration free energies (HFEs). No experimental data are fit during the development of our potentials; HFEs are only used to validate the quality of our models. AFM offers a unique strategy for fitting all FF parameters simultaneously to condensed phase forces computed with QM/MM. Furthermore, AFM generates unique interaction terms for each pair of atoms to describe non-bonded interactions instead of relying on combination rules. Our aim is to benchmark the use of AFM for small molecule HFE predictions based on MP2 gradient calculations. Other structural and thermodynamic quantities of our models will be investigated as further validation. This paper reports the development of force fields for dilute aqueous solutions of methane, ethane, methanol, and ethanol.

AFM has seen early success with modeling pure systems, such as water,^30,31^ graphene,^32^ and CO_2_.^33^ In terms of HFEs, AFM models for hydrated salts of simple ions have shown great success.^34,35^ However, relatively large HFEs are associated with the salts such that some degree of model imperfection only results in small percentage errors. The performance of AFM FFs on neutral solute HFE predictions has not been demonstrated. Prediction of HFEs of neutral molecules is challenging since such molecules tend to have much smaller HFEs when compared to the charged ones. The two hydrophobic alkanes in this study, methane and ethane, have experimental HFEs of only a few *RT*.^36^ For these solutes, the predicted HFEs would have to agree with experimental values within a fraction of *RT* to be deemed adequate.

The remainder of this paper is organized as follows: Sec. II gives an overview of the AFM algorithm, focusing on the details of the steps specific to these solutes; Sec. III describes simulation details for property calculations; Sec. IV discusses the performance of our solute models; and Sec. V concludes with a summary of our findings and an outlook for future FF development with AFM.

## II. DEVELOPMENT OF SOLUTE MODELS BY AFM

AFM is an iterative procedure that relies on “force matching” (FM) to parameterize potential energy functions to best reproduce forces from QM reference calculations. Detailed descriptions of the AFM procedure and its merits have been presented previously,^28,37^ and only a synopsis is given here. AFM iterates through three main steps: MD sampling of the phase space of interest, followed by the calculation of condensed phase reference forces with QM/MM, and finally, FM to re-parameterize the functional form of the FF. Together, these three steps comprise a single AFM “generation.” The parameterized FF from each generation is used for MD sampling in the next iteration, leading to improved sampling quality. After the fit converges, a few more generations of AFM are typically performed in order to generate a large training set of QM/MM reference forces. This large training set is referred to as the “global set,” and the fitting to such a global set allows better converged parameters for the final AFM FF.

While polarizable potentials are gaining well-deserved attention in recent years,^20,27,38,39^ we will focus on pairwise additive potentials in this work for their speed and support by most popular simulation packages. Only the solute–water intermolecular potentials and solute intramolecular terms will be created with AFM. The BLYPSP-4F model^29^ created previously by AFM will be used for water. This water model was fitted by AFM using coupled-cluster quality reference forces obtained with the Density Functional Theory with Supplemental Potential (DFT-SP) method^40^ and reproduces many properties in good agreement with experiments, such as radial distribution functions (RDFs), heat of vaporization, surface tension, dielectric constant, and diffusion constant.^30^ The specific procedure for developing the FF models for the solutes studied is detailed below.

### A. MD sampling step

For the MD sampling step, the initial guess FF was chosen to be OPLS-AA.^6^ OPLS-AA was used only for the first generation and was replaced by the AFM FF in subsequent generations. The periodic simulation box contains one solute molecule along with 266 waters for the case of methane or methanol and 342 waters for ethane or ethanol. Sampling was performed at 298.15 K and 1 bar for 6 ns with a 0.5 fs time step. The small time step was due to the use of a flexible water model. The temperature and pressure were maintained by using a Nosé–Hoover thermostat^41,42^ and Parrinello–Rahman barostat,^43,44^ with relaxation times of 2 ps and 5 ps, respectively. A cutoff of 9 Å was used for short-range nonbonded interactions with the long-range correction for both energy and pressure. The particle mesh Ewald (PME) method was used for long-range Coulombic interactions.^45^ The simulation was performed using GROMACS 2018.4.^46^ 100 configurations were extracted from the last 2 ns at equal intervals to be used in the QM/MM step.

### B. QM/MM reference forces step

The quality of the reference forces plays a major role in the predictive capabilities of an AFM model. Given the small HFEs of the solutes investigated in this work, any substantial error in reference forces could translate to an excessive percentage error in HFE. While DFT can be quite accurate, there is no consensus as to which functional would be the best in this context. Since only single point energy and gradient evaluations are required in AFM, a more reliable post-Hartree–Fock method can be used. To establish the optimal QM/MM method and basis set that offers sufficient accuracy at a moderate computational cost, potential energy surface (PES) scans were performed on selected dimer conformations for the methane–water system. The QM method and basis set identified to be the best for methane were also used for the other solutes in this study.

**FIG. 1.**
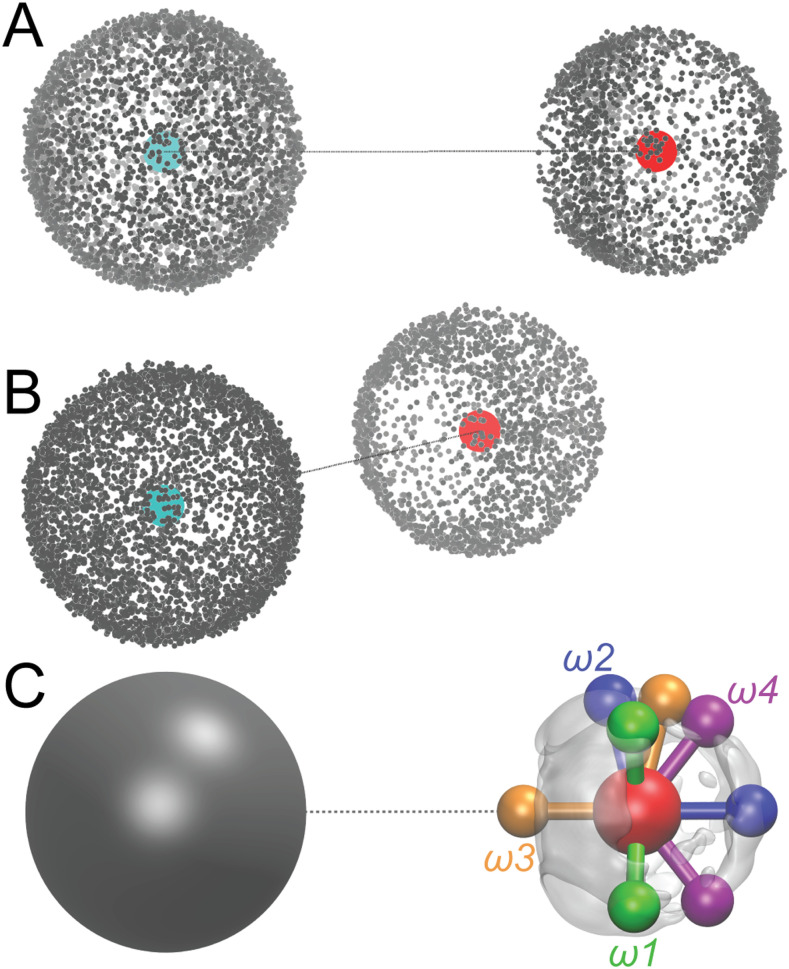
Distribution of hydrogens of methane (left) and of water molecules (right) for dimers extracted from a liquid simulation. Water has been translated to the same C–O distance without changing its relative orientation. Hydrogen atoms are depicted as gray, carbon is depicted as teal, and oxygen is depicted as red. (a) Methane appears isotropic, while water exhibits preferential hydrogen locations. (b) Rotated view showing the region of lowest hydrogen density around water oxygen, which is nearest to methane. (c) Methane hydrogen distribution depicted as purely isotropic, while distribution of water hydrogen represented by the transparent isosurface. Four proposed water orientations, *ω*1*–ω*4, are shown.

Since the goal of the final FF is to model hydrated methane, care was taken to include the most relevant dimer conformations for such PES scans. Methane and one of its nearest water molecules were exacted from uncorrelated conformations of the solution for a total of 1200 methane–water dimers. Only relative orientations are of interest since the intermolecular distances between the dimer were scanned. The dimers were rotated, and water was translated so that the methane carbon was always at the origin, and water oxygen was at (4, 0, 0). The resultant distribution of the density of the hydrogen atoms around methane and water is shown in Fig. 1. From Fig. 1, it can be seen that methane has a uniform angular distribution of hydrogen density, whereas there are preferential positions for the water hydrogen atoms, most likely a result of the hydrogen-bond networks between hydration water molecules.

Water orientations *ω*1 and *ω*2 were proposed so that the hydrogen positions reside in the hydrogen density maxima shown in Fig. 1, while orientations *ω*3 and *ω*4 were proposed without resorting to the hydrogen density distribution. To check the representativeness of the orientations proposed, the 1200 dimers extracted from the solution were classified into one of the four water orientations by minimizing the root mean square displacements (RMSDs) of the dimers relative to the proposed orientations. For the RMSD optimizations, the methane hydrogens were ignored, and only spin of the dimers along the C–O axis was allowed. From this analysis, it was found that configurations *ω*1 and *ω*2 represent ∼47% and ∼34% of the solution phase contact pairs, while configurations *ω*3 and *ω*4 represent only 17% and 2%, respectively. Therefore, the evaluation of the QM methods in this work focused on PES scans for orientations *ω*1, *ω*2, and *ω*3 only.

**FIG. 2.**
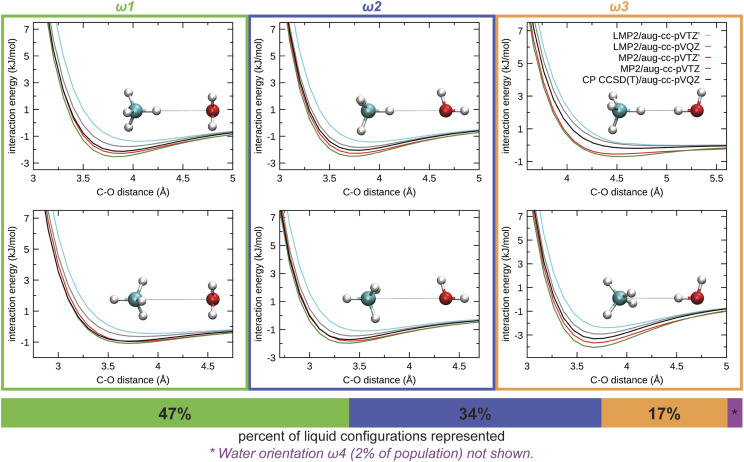
Potential energy scans along the C–O axis of representative methane–water dimer configurations. Scans are performed for two methane orientations for each water orientation, *ω*1, *ω*2, and *ω*3, as described in the text. Percentages of liquid population represented by each orientation of water are given by the bar below the plots.

PES scans along the intermolecular C–O distance computed with coupled-cluster singles, doubles, and perturbative triples [CCSD(T)], MP2,^47^ and local MP2 (LMP2)^48,49^ are shown in Fig. 2, using two orientations of methane for each water orientation, *ω*1, *ω*2, and *ω*3. The reference CCSD(T)/aug-cc-pVQZ scans were performed with the counter-poise (CP) correction.^50^ Density fitting^51,52^ was used for dimer energy computations for the scan. While performing CP corrections on dimers is straightforward, such corrections should be avoided for FM as many-body CP corrections are cumbersome and costly. Thus, the MP2 and LMP2 PESs examined for use with FM were computed without CP correction.

While it has been shown previously that LMP2 may help reduce the basis set superposition error (BSSE) in the reference forces,^33^ Fig. 2 shows that LMP2 overestimates repulsion between methane and water for all orientations, resulting in too shallow energy minima. When tested with MP2, the aug-cc-pVTZ basis set overbinds compared to the reference, most probably from BSSE. Overall, MP2 with the aug-cc-pVTZ′ basis set provides best agreement to the CCSD(T) reference. The aug-cc-pVTZ′ basis set was constructed by using the aug-cc-pVTZ basis set for the solute but removing the *f* functions from solvent water oxygen and *d* functions from water hydrogens. Thus, the MP2/aug-cc-pVTZ′ combination will be used for all QM/MM computations for all four molecules investigated. We acknowledge that better agreement of MP2/aug-cc-pVTZ′ with CP corrected CCSD(T) is probably partially due to a fortuitous cancellation of errors since MP2 with a full aug-cc-pVTZ basis set overbinds. It is also our assumption that if the MP2/aug-cc-pVTZ′ combination is sufficiently accurate for dimers, it will be similarly accurate for larger clusters, and the contribution from many-body effects will be properly accounted for.

To create the QM/MM conformations for the MP2 reference force calculations, the following procedure was used. For each of the 100 conformations saved in the MD step, the MM region was modeled with electrostatic embedding,^53^ using the charges of the BLYPSP-4F water model.^29^ The QM region was further divided into “fitting” and “buffer” regions, and only the forces on atoms in the fitting region were used for parameterization in the FM step. The buffer region shields the fitting region from the MM region to ensure that no atom being fit resides too close to a point charge. In each configuration, molecules were assigned to the fitting, buffer, or MM region via the following algorithm:

1. The solute and any water within 4.5 Å of a carbon atom or 3.6 Å of a solute oxygen atom will be referred to as the first hydration shell of the solute and included in the QM region.
2. The solute and three randomly selected water molecules in the first hydration shell comprise the fitting region.
3. Any water within 2.6 Å of any fitting region atom will be added to the QM region. All QM waters not in the fitting region will be the buffer region.
4. All water molecules not selected to be in the QM region compose the MM region.

Molecules in the fitting region are modeled with the aug-cc-pVTZ′ basis set. A smaller basis set is used for buffer region atoms to reduce electron spillover and basis set linear dependency: oxygen atoms in the buffer region will be modeled with the aug-cc-pVDZ basis set, whereas hydrogen will use the cc-pVDZ basis set. All QM/MM computations were performed with density fitting using the Molpro program.^54,55^

### C. FM step

As shown previously with AFM, it is challenging to fit dispersion interactions simultaneously with short-range repulsions,^28,29^ which are much stronger. We thus chose to fit the dispersion functions to energies from symmetry adapted perturbation theory (SAPT)^56,57^ prior to fitting other functions in the FF. SAPT is a double perturbation approach that permits decomposition of interaction energies into their components. SAPT E^(2)^ dispersion energy includes contributions from monomer correlations and is more accurate than dispersion from MP2. With the dispersion parameters determined by SAPT, each generation of AFM then follows a two-step FM procedure in which the partial charges and repulsion parameters were fitted in the first step and intramolecular parameters in the second step. The intermolecular functions were fitted to reproduce the net molecular forces and torques, while the intramolecular functions were fitted to the atomic forces.

**FIG. 3.**
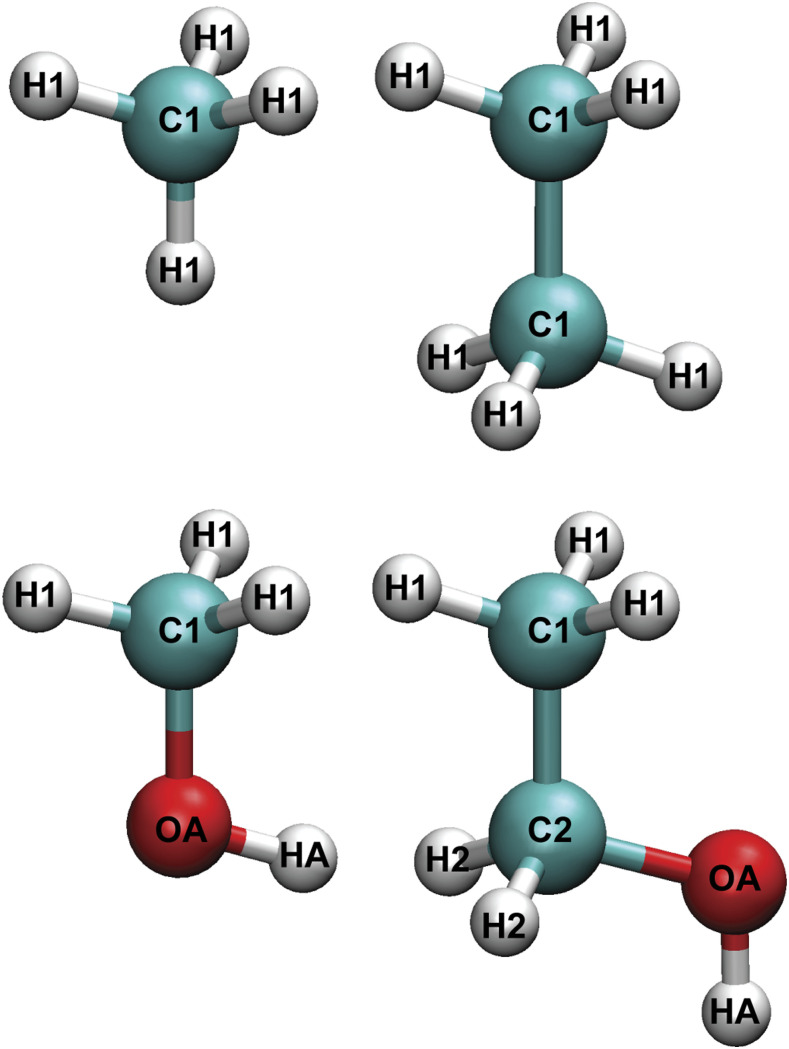
Atom types used for methane, ethane, methanol, and ethanol models. The same atom types in different molecules carry different parameters in our force fields.

The dispersion between solute and hydration water molecules was fitted to the following expression, placed between all solute heavy atoms and water oxygen (OW),

$$U_{disp}=\overset{M}{\underset{i=1}{\sum }}-\frac{C_{6,i-OW}}{r_{i-OW}^{6}},$$ (1)

where the sum is over the *M* heavy atoms of the solute and *C*_6_ are the fitting parameters. The atom-type naming convention used in this work is depicted in Fig. 3. We note that the same atom types carry different parameters in different molecules since we created a customized FF for each molecule instead of one generalized FF. SAPT E^(2)^ dispersion energies^57,58^ were computed with the aug-cc-pVTZ dimer-centered basis set for a minimum of 50 reference dimers for each solute–water system. These dimers were extracted from solution simulations with all molecules constrained to their gas phase MP2/aug-cc-pVDZ geometries. The distance between solute heavy atoms and water ranges from 7.5 Å to 12 Å for the extracted dimers, and care was taken to ensure a near-uniform distribution of the distances.

**FIG. 4.**
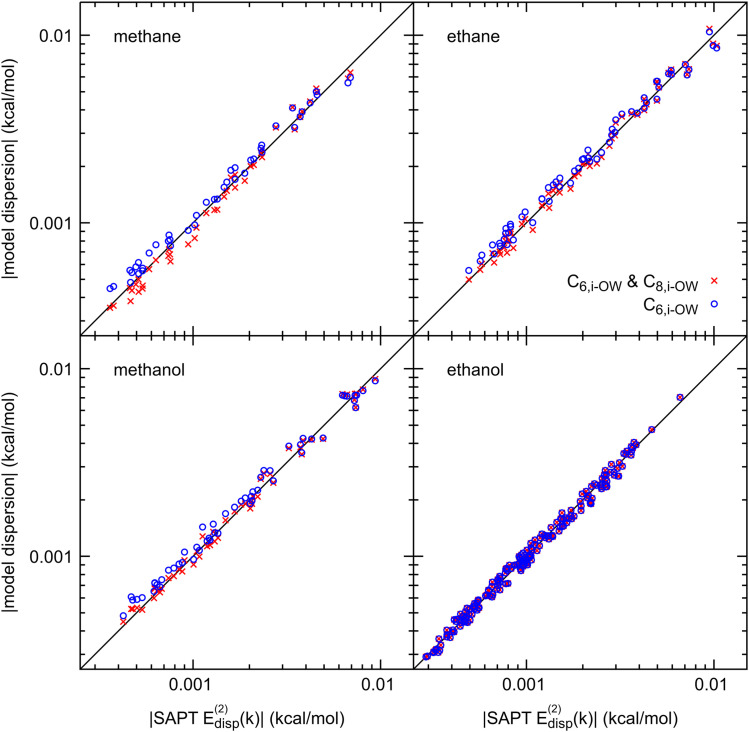
Scatter plot comparing fitted and SAPE E_2_ dispersion energies with and without the *C*_8_*/r*^8^ term. Inclusion of the *C*_8_*/r*^8^ term was not found to reduce the RMSE of the fit sufficiently to justify the inclusion in the FF’s dispersion model.

For each solute–water system, the contribution from higher order dispersion terms was also explored. Figure 4 shows the fitted dispersion with and without the inclusion of additional $-C_{8,i-OW}/r_{i-OW}^{8}$ terms between heavy atoms. Methane shows the maximum benefit from the higher order term, where this term reduces the dispersion energy root mean square error (RMSE) by 10%. For larger molecules, such as ethanol, no reduction in RMSE can be seen with up to four significant numbers. We decided to only keep the 1/*r*^6^ term for modeling dispersion for all solutes.

After the dispersion terms were determined, the two-step FM was carried out by the CRYOFF program, version 2.7.2.3b,^59^ developed in the Wang lab. Point charges are placed on every atom, and the solute is constrained to be neutral. The repulsion is modeled by a set of exponential expressions,

$$U_{repul}=A_{ij}e^{-\alpha_{ij}r_{ij}},$$ (2)

where *r*_*ij*_ is distances between atoms *i* and *j*. All repulsion and Coulombic parameters are fitted simultaneously.

Several possibilities exist for the choice of exponential repulsion terms between each solute and water. In the supplementary material, we summarize the RMSEs for some of these possible choices for methane and methanol. For methane, the placement of repulsion between the aliphatic hydrogen (H1) and water hydrogen (HW) leads to an RMSE lower than a repulsion between carbon (C1) and HW by 0.001 kcal/(mol Å). Using both the H1–HW and C1–HW repulsions—thus introducing two additional parameters—only led to a further reduction in the RMSE by 0.0001 kcal/(mol Å). Therefore, only the H1–HW term was kept for the alkanes. For methanol, it was concluded that keeping the H1–OW repulsion is unnecessary and repulsion between hydroxyl hydrogen (HA) and OW should be included. The final sites for the placement of repulsions are summarized along with the other intermolecular parameters in Table I.

**TABLE I.** Intermolecular potential parameters of the methane, ethane, methanol, and ethanol models.

		Solute			
Atom type(s)	Parameter (units)	Methane	Ethane	Methanol	Ethanol
Coulombic: $U_{Cou}\left(r_{ij}\right)=q_{i}q_{j}/\left(4\pi \epsilon_{0}r_{ij}\right)$					
C1	*q*_*i*_ (e)	−0.4414	−0.1581	0.5845	−0.1170
H1	*q*_*i*_ (e)	0.1103	0.05271	−0.0857	0.01916
OA	*q*_*i*_ (e)			−0.7606	−0.8139
HA	*q*_*i*_ (e)			0.4332	0.4545
C2	*q*_*i*_ (e)				0.5176
H2	*q*_*i*_ (e)				−0.0493
Dispersion: $U_{disp}\left(r_{i-OW}\right)=-C_{6,i-OW}/r_{i-OW}^{6}$					
C1–OW	*C*_6*,i-OW*_ (kcal Å^6^/mol)	1 246.03	1 051.53	1 133.02	1 042.49
OA–OW	*C*_6*,i-OW*_ (kcal Å^6^/mol)			532.66	536.91
C2–OW	*C*_6*,i-OW*_ (kcal Å^6^/mol)				743.17
Repulsion: $U_{repul}\left(r_{ij}\right)=A_{ij}e^{-\alpha_{ij}r_{ij}}$					
H1–OW	*A*_*ij*_ (kcal/mol)	12 897.527	5 858.829		
	*α*_*ij*_ (Å^−1^)	4.2262	3.7471		
H1–HW	*A*_*ij*_ (kcal/mol)	847.661	2 470.127	3 434.019	4 417.596
	*α*_*ij*_ (Å^−1^)	3.7760	4.2923	4.0562	4.3219
C1–OW	*A*_*ij*_ (kcal/mol)	421 840.024	2 431 164.300	2 478 705.652	2 823 170.400
	*α*_*ij*_ (Å^−1^)	4.1043	4.7671	4.6971	4.6294
HA–OW	*A*_*ij*_ (kcal/mol)			1 120.694	760.790
	*α*_*ij*_ (Å^−1^)			3.7279	3.3417
OA–HW	*A*_*ij*_ (kcal/mol)			1 244.524	1002.112
	*α*_*ij*_ (Å^−1^)			3.6871	3.3996
OA–OW	*A*_*ij*_ (kcal/mol)			386 285.956	265 074.350
	*α*_*ij*_ (Å^−1^)			4.3299	4.1731
C2–OW	*A*_*ij*_ (kcal/mol)				19 425 485.000
	*α*_*ij*_ (Å^−1^)				5.5968
H2–OW	*A*_*ij*_ (kcal/mol)				9426.774
	*α*_*ij*_ (Å^−1^)				4.1145
H2–HW	*A*_*ij*_ (kcal/mol)				2939.827
	*α*_*ij*_ (Å^−1^)				4.3131

Since our largest solute molecule is ethanol, no intramolecular non-bonded interactions are included. All covalently bonded atoms will have a harmonic bond term, and every three atoms connected by two covalent bonds have a harmonic angle term. Inclusion of higher order polynomials for neither bond nor angle terms was found to significantly reduce the RMSE of the fit.

Dihedral angles in these FFs are modeled by the cosine function,

$$U_{dih}\left(\phi \right)=k_{dih}\left(1+\cos\left(3\phi -\phi_{e}\right)\right),$$ (3)

where *ϕ* is the torsional angle, *k*_*dih*_ is the fitting parameter, and *ϕ*_*e*_ is fixed to zero. It can be shown that for every two covalently bonded sp^3^ atoms, only one degree of freedom exists for torsional motion, once bond and angle degrees of freedoms are accounted for. Thus, we only fit the H1–C1–C1–H1 torsional term for ethane, H1–C1–OA–HA for methanol, and H1–C1–C2–OA and C1–C2–OA–HA terms for ethanol.

To reduce fluctuation in parameters, reference forces from the current and preceding generation’s QM/MM steps were fitted together starting from the second generation, for a total of 200 QM/MM configurations contributing to each FM step. At least seven AFM generations were carried out for each solute, with the last five generations fitted together as the global fit.

## III. COMPUTATIONAL DETAILS FOR PROPERTY CALCULATIONS

To validate the FFs developed and gain insight into the hydration of the small solute molecules studied, HFEs, enthalpies of hydration, diffusion constants, radial distribution functions (RDFs), and power spectra were computed and compared with experimental data where available.

For the HFE determination, alchemical simulations were performed using the Bennett acceptance ratio (BAR) method^60,61^ as implemented in GROMACS. Alchemical annihilation of each solute was conducted by first removing Coulombic and, subsequently, short-range non-bonded interactions between the solute and water. For the alcohols, Coulombic interactions were removed in 11 steps, followed by removing short-range interactions in 10 steps. For the alkanes, Coulombic interactions were removed in only six steps, followed by 10 steps to remove the short-range interactions. Only one solute was present in each simulation box, along with 266 water molecules. For ethane and ethanol, the HFE was also measured with a 749 water box and is reported in the supplementary material. The measured HFE is very close to the value obtained with the 266 water box, showing negligible finite size effects.

A soft-core potential was applied to the solute–solvent short-range nonbonded interaction in order to avoid numerical instability caused by particle overlaps when such interactions are being removed.^46,62^ All alchemical simulations used a soft-core power of 1, a soft-core radius of 2.65 Å, and a value of 1.0 for the *α* parameter in Ref. 62. It is noteworthy that the use of exponential repulsion leads to less numerical instability when compared to the 1/r^12^ repulsion used in many other FFs.

Each window of the alchemical simulation was sampled from a 10 ns trajectory following a 400 ps equilibration period. For the simple solutes studied in this work, 400 ps should be sufficient for equilibration.^63^ All alchemical simulations were performed at 298 K and 1 bar with a stochastic Langevin integrator^64^ for temperature control and a Parrinello–Rahman barostat with a 5 ps relaxation time for pressure. Short-range non-bonded interactions were truncated at 9 Å, with long-range corrections for energy and pressure applied. Electrostatics were handled with PME.^45^

While nonideality of the solute in the gas phase has been shown to be important for some systems,^65^ such contributions are not expected to be significant at ambient temperature for the solute molecules studied in this work.

The enthalpies of hydration were computed from ensemble averages obtained by MD simulations, according to the following formula:^10,66^

$$\begin{array}{ccc} \Delta H_{solution} & = & {\left\langle E\right\rangle }_{sol}-\left({\left\langle E\right\rangle }_{water}+{\left\langle E\right\rangle }_{solute}\right)\\ & & +\;P{\left\langle V\right\rangle }_{sol}-P{\left\langle V\right\rangle }_{water}-RT, \end{array}$$ (4)

where $\left\langle E\right\rangle$ is the average internal energy of the solution (sol), water, and solute, determined from separate simulations. 20 independent, 10 ns trajectories were simulated at 298 K and 1 bar to obtain the average internal energies for the solutions and water. Only 266 water molecules were used to solvate each solute to reduce the variance of the internal energy measurements. The solute simulations were performed with only one molecule in the gas phase.

Diffusion coefficients were calculated by using the Einstein equation.^67^ Cubic boxes containing one solute and 749 waters were used. 10 evenly spaced configurations were obtained from the final 500 ps of a 3 ns simulation at 298 K and 1 bar. From each of these 10 configurations, 3 ns trajectories were simulated at 298 K in the canonical ensemble with random initial velocities^68^ at the average volume for 1 bar. A Nosé–Hoover thermostat with a relaxation time of 5 ps was used to control temperature. The mean square displacement (MSD) was fit in the range of 10 ps–40 ps, and error bars were determined as the standard error of the mean. Solute–water RDFs were computed using 200 000 snapshots from 10 ns NPT trajectories.

The power spectra provide a good estimate of the vibrational spectra of the system. The power spectrum of each solute was computed from the velocity autocorrelation function of a 500 ps trajectory in a cubic box of 266 waters. These trajectories were simulated with a 0.1 fs time step, saving every 0.2 fs.

## IV. RESULTS AND DISCUSSION

The parameters for the intermolecular and intramolecular terms of the solute FFs from AFM are reported in Tables I and II, respectively. A key validation of the quality of these AFM models is their ability to predict HFEs. Theoretical prediction of small molecule HFE has been a long sought-after goal, with many methods devoted to this subject.^69,70^

**TABLE II.** Intramolecular potential parameters of methane, ethane, methanol, and ethanol models.

		Solute			
Atom type	Parameter (units)	Methane	Ethane	Methanol	Ethanol
Bonds: $U_{bon}\left(r\right)=k_{bon}{\left(r-r_{e}\right)}^{2}$					
C1–H1	*r*_*e*_ (Å)	1.0902	1.0931	1.0914	1.0924
	*k*_*bon*_ (kcal/mol)	765.7422	734.1599	742.2292	744.9736
C1–C1	*r*_*e*_ (Å)		1.5301		
	*k*_*bon*_ (kcal/mol)		541.1053		
C1–OA	*r*_*e*_ (Å)			1.4290	
	*k*_*bon*_ (kcal/mol)			604.7620	
OA–HA	*r*_*e*_ (Å)			0.9597	0.9609
	*k*_*bon*_ (kcal/mol)			1080.0477	1056.6764
C1–C2	*r*_*e*_ (Å)				1.5204
	*k*_*bon*_ (kcal/mol)				551.9110
C2–H2	*r*_*e*_ (Å)				1.0937
	*k*_*bon*_ (kcal/mol)				742.5561
C2–OA	*r*_*e*_ (Å)				1.4396
	*k*_*bon*_ (kcal/mol)				577.2346
Angles: $U_{ang}\left(\theta \right)=k_{ang}{\left(\theta -\theta_{e}\right)}^{2}$					
H1–C1–H1	*θ*_*e*_ (deg)	102.396	100.053	107.854	101.371
	*k*_*ang*_ (kcal/mol)	76.560	71.451	72.459	73.271
H1–C1–C1	*θ*_*e*_ (deg)		105.272		
	*k*_*ang*_ (kcal/mol)		96.802		
H1–C1–OA	*θ*_*e*_ (deg)			108.928	
	*k*_*ang*_ (kcal/mol)			112.625	
C1–OA–HA	*θ*_*e*_ (deg)			109.098	
	*k*_*ang*_ (kcal/mol)			96.108	
H1–C1–C2	*θ*_*e*_ (deg)				104.971
	*k*_*ang*_ (kcal/mol)				96.623
C1–C2–H2	*θ*_*e*_ (deg)				107.711
	*k*_*ang*_ (kcal/mol)				97.955
C1–C2–OA	*θ*_*e*_ (deg)				109.110
	*k*_*ang*_ (kcal/mol)				167.060
H2–C2–H2	*θ*_*e*_ (deg)				103.715
	*k*_*ang*_ (kcal/mol)				71.873
C2–OA–HA	*θ*_*e*_ (deg)				109.167
	*k*_*ang*_ (kcal/mol)				99.632
H2–C2–OA	*θ*_*e*_ (deg)				105.522
	*k*_*ang*_ (kcal/mol)				110.565
Dihedrals: $U_{dih}\left(\phi \right)=k_{dih}\left(1+\cos\left(3\phi -\phi_{e}\right)\right)$					
H1–C1–C1–H1	*ϕ*_*e*_ (deg)		0		
	*k*_*dih*_ (kcal/mol)		0.4404		
H1–C1–OA–HA	*ϕ*_*e*_ (deg)			0	
	*k*_*dih*_ (kcal/mol)			0.2637	
H1–C1–C2–OA	*ϕ*_*e*_ (deg)				0
	*k*_*dih*_ (kcal/mol)				0.4470
C1–C2–OA–HA	*ϕ*_*e*_ (deg)				0
	*k*_*dih*_ (kcal/mol)				0.4837

The experimental HFEs for gases with low solubilities, such as methane and ethane, are generally obtained by measuring the concentration of dissolved gas in equilibrium with excess solute in the gaseous state. The concentration of dissolved gas can be deduced by the volume of gas absorbed^71,72^ or by head-space chromatography.^73–75^ For methanol and ethanol, the HFE can be calculated by Henry’s law constants, which is the ratio of equilibrium vapor pressure and solute mole fraction at low concentration. The vapor pressure can be measured with an isoteniscope.^76,77^ It appears that Ben-Naim’s estimates of the methanol and ethanol HFEs were obtained by the difference of free energies of the formation of aqueous and gas phase solutes in their respective standard states from the National Bureau of Standards tables.^36,78^

Variance is observed in the experimental HFEs measured by different groups. Such variance has been attributed to various experimental challenges,^79^ such as the need to ensure equilibrium between the gas and solution phases,^72^ avoiding either oversaturation or undersaturation. Other factors, such as losses in mass balance and difficulties in measuring concentrations in dilute solutions,^80^ also lead to uncertainties.^81^

Table III reports the HFE of each of the four solutes computed with BAR, using the FFs created by AFM with the MP2/aug-cc-pVTZ′ reference. Excellent agreement can be observed for all four solutes with the RMSE being 1.0 kJ/mol when Ben-Naim’s references are used as the standard. Compared to Ben-Naim’s HFEs, the largest error is observed for ethane, which is 1.6 kJ/mol. For methanol and ethanol, Henry’s law estimates compiled by Sander show a span of 3 kJ/mol and 2 kJ/mol, respectively. The differences between AFM and experimental HFEs are thus comparable to the differences between experimental estimates. The AFM-based estimate of −20.45 kJ/mol for ethanol is actually right in the middle of the largest and smallest experimental Henry’s law estimates. Chemical accuracy is generally considered to be 1 kcal/mol, and the 1.0 kJ/mol RMSE of our predictions is ∼0.25 kcal/mol, showing that these AFM-based predictions of HFEs achieve chemical accuracy when MP2 is used to provide reference gradients.

**TABLE III.** Predicted and experimental HFEs of the four solutes studied in this work. Experimental values reported as “Sander” are derived from Sander’s compilation of Henry’s law constants, *H*_*cp*_.^85^ The Sander compilation contains multiple *H*_*cp*_ for each solute; thus, only the smallest and the largest HFEs calculated from experimental measurements within his data set are shown. All values are in kJ/mol.

	Simulated			Expt.	
Solute	AFM	CGenFF^84^	GAFF^10^	Ben-Naim^36^	Sander^a^ smallest, largest
Methane	9.31 ± 0.02	9.20 ± 0.12	10.25 ± 0.04	8.39	8.3, 8.7
Ethane	9.27 ± 0.03	8.38 ± 0.12	10.29 ± 0.04	7.67	7.4, 7.6
Methanol	−21.91 ± 0.08	−18.57 ± 0.12	−14.60 ± 0.08	−21.34	−21.7, −18.7
Ethanol	−20.45 ± 0.07	−18.87 ± 0.12	−14.18 ± 0.08	−21.13	−21.4, −19.6

^a^ Computed from the compiled set of experimental *H*_*cp*_ data, Ref. 85, via −*RT*·ln(*RTH*_*cp*_).

For these molecules, HFEs based on CGenFF^82^ and GAFF^5,83^ are also reported. CGenFF and GAFF are the small molecule general FFs for CHARMM and AMBER, respectively. Both the CGenFF and GAFF HFEs were measured in TIP3P water. The CGenFF HFEs^84^ are in better agreement with experimental numbers than the GAFF HFEs^10^ for these molecules, which may be partly due to the explicit fitting of small molecular–water interactions in the development of CGenFF. The AFM-based models, along with CGenFF and GAFF, provide good predictions of the HFEs of methane and ethane. However, for methanol and ethanol, GAFF seems to be significantly worse. The AFM predictions are probably better than CGenFF for these alcohols, although the difference between the AFM and CGenFF predictions is comparable to the variance in experimental HFEs based on Henry’s law constants from different measurements. Even though the AFM models are not performing much better than CGenFF or GAFF for methane and ethane, we still consider these predictions a success since no experimental data were ever fitted in the development of our models.

It is interesting to note that the HFE of methane is close to that of ethane, and the HFE of methanol is close to that of ethanol. Thus, the addition of a methyl group did not change the HFE by more than 1 kJ/mol. The additional methyl group is expected to increase the cavitation energy; consequently, one would anticipate a more positive HFE for the larger solutes.

To better understand the contribution to HFE from the additional methyl group, alchemical simulations were performed in which only the repulsive part of the short-range non-bonded interactions is switched on to compute the cavitation energy. The dispersion contribution to the HFE is then estimated by subtracting the cavitation energy from the contribution to HFE from switching on all the short-range non-bonded interactions. The Coulombic contribution was computed by switching on Coulombic interactions in the presence of the short-range non-bonded terms between the solute and water, as discussed previously in Sec. II A.

The contribution to the total HFE from different components is shown in Table IV. It is not surprising that the Coulombic part has almost no contribution to the overall HFE for methane and ethane. The Coulombic contribution for ethanol is 4 kJ/mol larger than that of the methanol, which can be explained by its larger dipole moment. The cavitation energy is indeed larger for ethane and ethanol, with the additional methyl group contributing about 11 kJ/mol in both cases. However, for ethane, the increased dispersion contribution compensates for the increased cavitation energy. In the case of ethanol, the increased dispersion and Coulombic contributions compensate for the larger cavitation energy, leading to a very small change in HFE upon the addition of an extra methyl group.

**TABLE IV.** Computed HFEs and contributions from different FF components of the AFM models. All values are in kJ/mol.

Solute	HFE	Coulombic	Repulsion	Dispersion	Total short range
Methane	9.31 ± 0.02	−0.25 ± 0.01	36.75 ± 0.16	−27.19 ± 0.16	9.56 ± 0.02
Ethane	9.27 ± 0.03	−0.03 ± 0.01	48.06 ± 0.12	−38.76 ± 0.12	9.30 ± 0.03
Methanol	−21.91 ± 0.08	−27.30 ± 0.01	41.23 ± 0.21	−35.84 ± 0.22	5.39 ± 0.08
Ethanol	−20.45 ± 0.07	−31.27 ± 0.01	52.76 ± 0.16	−41.94 ± 0.17	10.82 ± 0.07

Hydration enthalpies computed for each solute are reported in Table V, along with experimental references from three different sources. Excellent agreement can be seen for all molecules with the largest deviation being 2.5 kJ/mol, which is for methane when compared to the Ben-Naim reference value. At the same time, the difference between Ben-Naim and Cabani’s experimental values is around 2.0 kJ/mol–2.5 kJ/mol for each solute.

**TABLE V.** Enthalpies of hydration at infinite dilution, 1 bar, and 298 K. All values are in kJ/mol.

		Expt.		
Solute	AFM^a^	Ben-Naim^36^	Cabani^86^	CRC^87^
Methane	−8.8 ± 0.7	−11.49	−13.79	−12.0
Ethane	−15.4 ± 0.6	−17.46	−19.76	−17.9
Methanol	−43.6 ± 0.8	−42.89	−44.52	−45.1^b^
Ethanol	−48.9 ± 0.8	−50.42	−52.40	−50.6

^a^ Error estimates are the standard of the mean from 20 simulations of 10 ns each.

^b^ The CRC handbook lists −52.0 kJ/mol. This value is likely to be a transcription error, as CRC cites the work of Plyasunov *et al.* for the hydration enthalpy of methanol. The value of Plyasunov and Shock from Ref. 88 is reported here.

Diffusion coefficients for each solute in BLYPSP-4F water are shown in Table VI. Graphs reporting MSD as a function of time are shown in the supplementary material. The BLYPSP-4F water model has a diffusion constant of 2.46 × 10^−5^ cm^2^/s,^89^ which is slightly larger than the experimental value of 2.3 × 10^−5^ cm^2^/s. A solute cannot diffuse without displacing water molecules; thus, the diffusion constant of the solute is affected by the diffusion of water. When compared to the available experimental data, it is clear that the FFs developed with AFM give good estimates for diffusion constants. The computed diffusion constants in this work have not been corrected for finite size effects^90^ or quantum nuclear effects.^91^ The finite size correction should be small since a fairly large 749 water box was used. However, some ambiguity still remains for a direct comparison between simulated and experimental diffusion constants. It is worth noting, though, that the difference between the experimental and model diffusion coefficients is comparable or even smaller than the variation among different experimental measurements except for ethanol.

**TABLE VI.** Diffusion coefficients of aqueous solutes at infinite dilution, at 298 K, multiplied by 10^5^. All values are reported in cm^2^/s. Errors on predicted values reported as the standard error of the mean of 10 independent simulations.

Solute	AFM	Expt.			
Methane	1.79 ± 0.05	1.81^92^	1.88^93^	1.49^94^	1.49^95^
Ethane	1.28 ± 0.03	1.52^92^	1.52^93^	1.20^95^	
Methanol	1.50 ± 0.05	1.56^96^	1.56^97^	1.51^98^	
Ethanol	1.32 ± 0.03	1.24^97^	1.24^94^	1.23^98^	

**FIG. 5.**
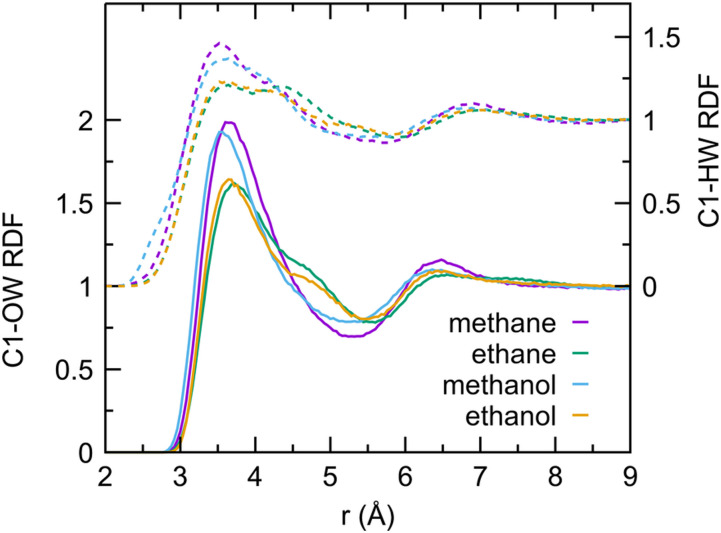
Radial distribution functions of water hydrogen (dashed lines and right ordinate axis) and water oxygen (solid lines and left ordinate axis) around the aliphatic carbons of AFM methane, ethane, methanol, and ethanol.

**FIG. 6.**
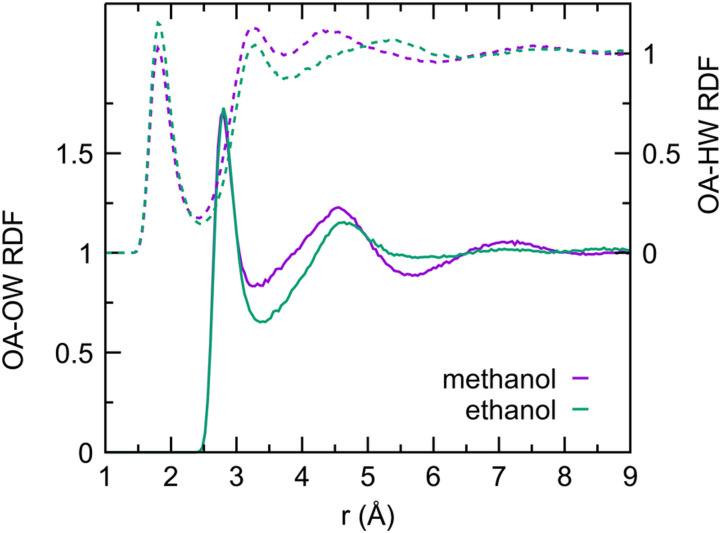
Radial distribution functions of water hydrogen (dashed lines and right ordinate axis) and water oxygen (solid lines and left ordinate axis) around the hydroxyl oxygen of AFM methanol (purple curves) and ethanol (green curves).

**FIG. 7.**
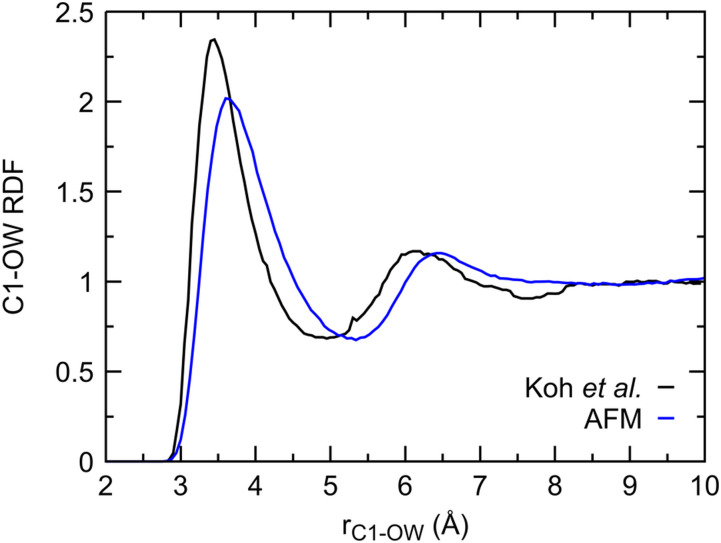
Comparison of experimental (black) and computed (blue) methane–water RDFs. Both RDFs are measured at 145 bars and 291 K.

**FIG. 8.**
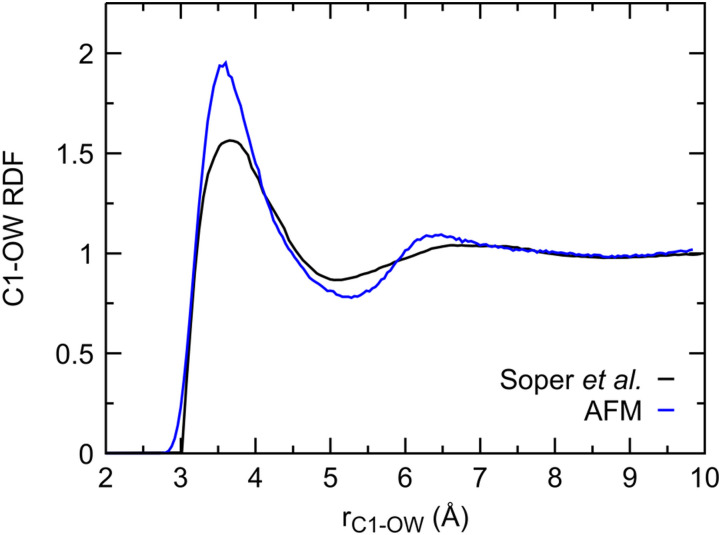
Comparison of experimental (black) and computed (blue) methanol–water RDFs. Both RDFs are measured near 1 bar at 293 K.

Different solute–water RDFs are shown in Figs. 5–8. Figure 5 shows the structure of water around the aliphatic carbon of each solute. For the first hydration shell, the water hydrogen is slightly closer than the aliphatic carbon by 0.1 Å–0.2 Å than the water oxygen, consistent with the dominance of orientations *ω*1 and *ω*2 shown in Fig. 1. In these orientations, water maximizes hydrogen bonds with other waters in the presence of an aliphatic carbon, which is hydrophobic. A second hydration shell peak around 6.4 Å can be clearly seen. It is interesting to note that, on average, the water hydrogen is further away from the water oxygen in the second hydration shell. Figure 6 characterizes the hydration structure around the hydroxyl oxygen in methanol and ethanol. Not surprisingly, a distinct structuring of water can be seen with the first hydrogen peak 1 Å closer than the first water oxygen peak at 2.8 Å. The second hydrogen peak at 3.3 Å is most likely formed by the other hydrogen of the first hydration shell waters. It is interesting to note that, while third hydration shell peaks cannot be seen around the ethanol hydroxyl oxygen, a peak consistent with a third hydration shell can be seen around the methanol hydroxyl oxygen.

Experimental partial RDFs of the solutes in dilute solutions are not readily available, largely due to their difficulty to be separated from those of neat water at low concentrations. One of the few experimental RDFs we identified in the literature is for 2.6 mol. % methane at 145 bars and 291 K.^99^ The AFM RDF is measured with only one methane in the box and, thus, corresponds to infinite dilution. At the higher concentration, the experimental RDF has a tighter and more structured first hydration shell (Fig. 7). This is not surprising considering that the study of Koh *et al.* was in the context of water structuring during the formation of methane clathrate. The conditions of the experimental data shown in Fig. 7 are less than 2 K from the methane–clathrate stability line, which, along with the higher concentration of methane, might have prompted the increased structuring of water molecules.

When our AFM methanol is compared to a 10 mol. % methanol solution at 293 K,^100^ the positions of the RDF peaks are in good agreement (Fig. 8). The increased height of the RDF peaks of our methanol model can be intuitively understood from the fact that our dilute simulation has a significantly higher water concentration.

**FIG. 9.**
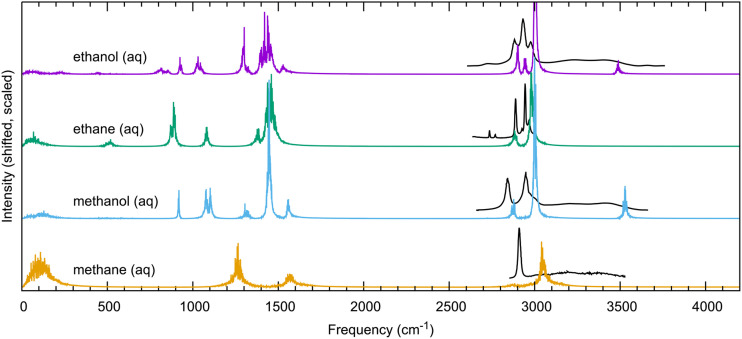
Vibrational spectra of all aqueous solutes. Frequency scaling corrections for MP2/aug-cc-pVTZ were applied to all AFM spectra according to the literature.^101^ Experimental Raman spectra are shown in black for methane,^102^ methanol,^103^ ethane,^104^ and ethanol.^103^ Peak intensities have been scaled arbitrarily to aid viewing.

The power spectrum of each solute in aqueous solution is shown in Fig. 9. Although power spectra do not obey selection rules, they will provide a reasonable estimate of experimental vibrational frequencies. Vibrational frequencies computed with an electronic structure method are commonly scaled to achieve better agreement with experiments.^101^ The scaling factor depends on the electronic structure method and partly accounts for quantum nuclear effects and neglect of bond anharmonicity. For MP2/aug-cc-pVTZ, it has been suggested by Merrick *et al.* to scale the high and low frequencies by 0.9598 and 1.012, respectively.^101^ These scaling factors have been applied to all four simulated spectra shown in Fig. 9.

The experimental spectra for the four solutes are shown for the region above 2500 cm^−1^.^102–104^ It is clear from Fig. 9 that for ethane and ethanol, the C–H stretch modes between 2800 cm^−1^ and 3100 cm^−1^ are in good agreement with experiments. The alcohols’ O–H stretch near 3500 is not clearly visible from experiments since it couples strongly with the O–H stretch in water. Only the power spectrum of the solute is reported in Fig. 9; thus, the O–H stretch stands out clearly without any background due to water. For methane and methanol, the power spectra from classical MD simulations predict a blueshift of the C–H stretch modes by 100 cm^−1^–150 cm^−1^ even with the scaling factor. We anticipate that for such molecules, explicit treatment of quantum nuclear effects could be important and might be responsible for the disagreement in the vibrational frequencies.

## V. CONCLUSIONS

Models of four non-electrolyte solutes, methane, ethane, methanol, and ethanol, in dilute aqueous solutions were developed by force matching MP2 gradients with AFM. The ability of these FFs to predict HFEs is of particular interest due to the HFE being an important fundamental property. These solutes were chosen partly because experimental HFEs are readily available by which to gauge the quality of the AFM potentials. Without fitting to any experimental data, all four HFEs are in excellent agreement with experiments, showing better performance than CGenFF and GAFF for methanol and ethanol. For methane, ethane, methanol, and ethanol, the AFM-based predictions achieved an RMSE of less than 0.25 kcal/mol relative to experimental data from Ben-Naim, thus reaching chemical accuracy.

Enthalpies of hydration, diffusion coefficients, radial distribution functions, and power spectra of the AFM-based models are also computed with classical MD. Excellent agreement is achieved for enthalpies of hydration and diffusion constants, further validating the quality of our models. It is worth noting that the AFM models were not biased to predict any properties more accurately than others, and some disagreement between the experimental and simulated results is likely due to limitations in the classical MD simulations. The ability of the AFM FFs to make high quality predictions for many properties suggests that MP2 with an aug-cc-pVTZ quality basis set well approximates the true Born–Oppenheimer PES for the hydration of these solutes.

Although these solutes are fairly simple, they represent both molecules with small solubilities and those indefinitely miscible with water. Cases such as methane and ethane provide a strong test for the quality of the models since minor disagreement in predicted HFEs can lead to large relative errors when the absolute HFE is small. This work shows the promise of AFM in blind predictions of HFEs. Further work on the application of AFM to more complex solute molecules is worthy of pursuit.

## SUPPLEMENTARY MATERIAL

See the supplementary material for RMSEs of different choices of repulsion potentials for methane and methanol, finite size effect testing for HFE, assessment of SAPT dispersion energy fitting models, and figures of MSD.

## Data Availability

The force fields and reference data files for fitting the force fields can be found at https://wanglab.uark.edu/Models. The remaining data that support the findings of this study are available from the corresponding author upon request.
